# Reductive dehalogenase of *Dehalococcoides mccartyi* strain CBDB1 reduces cobalt- containing metal complexes enabling anodic respiration

**DOI:** 10.3389/fmicb.2024.1457014

**Published:** 2024-10-23

**Authors:** Marie Eberwein, Nadine Hellmold, Ronny Frank, Darja Deobald, Lorenz Adrian

**Affiliations:** ^1^Department Molecular Environmental Biotechnology, Helmholtz Centre for Environmental Research – UFZ, Leipzig, Germany; ^2^Centre for Biotechnology and Biomedicine, Biochemical Cell Technology, Leipzig University, Leipzig, Germany; ^3^Department of Geobiotechnology, Institute of Biotechnology, Technische Universität Berlin, Berlin, Germany

**Keywords:** organohalide respiration, mediated extracellular electron transfer, EET, bioelectrochemical cultivation, cobalt chelates, energy conservation, proton motive force (*pmf*)

## Abstract

Microorganisms capable of direct or mediated extracellular electron transfer (EET) have garnered significant attention for their various biotechnological applications, such as bioremediation, metal recovery, wastewater treatment, energy generation in microbial fuel cells, and microbial or enzymatic electrosynthesis. One microorganism of particular interest is the organohalide-respiring bacterium *Dehalococcoides mccartyi* strain CBDB1, known for its ability to reductively dehalogenate toxic and persistent halogenated organic compounds through organohalide respiration (OHR), using halogenated organics as terminal electron acceptors. A membrane-bound OHR protein complex couples electron transfer to proton translocation across the membrane, generating a proton motive force, which enables metabolism and proliferation. In this study we show that the halogenated compounds can be replaced with redox mediators that can putatively shuttle electrons between the OHR complex and the anode, coupling *D. mccartyi* cells to an electrode *via* mediated EET. We identified cobalt-containing metal complexes, referred to as cobalt chelates, as promising mediators using a photometric high throughput methyl viologen-based enzyme activity assay. Through various biochemical approaches, we show that cobalt chelates are specifically reduced by CBDB1 cells, putatively by the reductive dehalogenase subunit (RdhA) of the OHR complex. Using cyclic voltammetry, we also demonstrate that cobalt chelates exchange electrons with a gold electrode, making them promising candidates for bioelectrochemical cultivation. Furthermore, using the AlphaFold 2-calculated RdhA structure and molecular docking, we found that one of the identified cobalt chelates exhibits favorable binding to RdhA, with a binding energy of approximately −28 kJ mol^−1^. Taken together, our results indicate that bioelectrochemical cultivation of *D. mccartyi* with cobalt chelates as anode mediators, instead of toxic halogenated compounds, is feasible, which opens new perspectives for bioremediation and other biotechnological applications of strain CBDB1.

## 1 Introduction

The conversion of energy stored in the redox potential difference between two redox pairs into a biologically usable form, such as a proton motive force (*pmf*) or ATP, represents the primary function of respiratory chains and holds significant promise for bioremediation and microbial electrosynthesis. Respiratory chains are fine-tuned assemblies of membrane-bound, redox-active proteins that catalyze redox reactions and couple electron flow to proton translocation across the cytoplasmic membrane against an electrochemical gradient (Marreiros et al., 2016). Extracellular electron transfer (EET) is one type of microbial respiration facilitating electron transfer between microbial cells and extracellular materials, including naturally-occurring metal compounds and artificial electrodes (Kato, 2015). Two primary modalities lie at the core of microbial EET mechanisms: direct EET and mediated EET.

Direct EET involves direct physical contact between microbial cells and insoluble electron acceptors or donors, facilitating electron exchange across the cellular membrane (Kato, 2015). Investigations into direct EET, particularly in model organisms like the iron-reducing bacteria *Geobacter sulfurreducens, Shewanella oneidensis*, and the iron-oxidizing bacterium *Acidithiobacillus ferrooxidans*, have revealed the crucial involvement of redox-active proteins, primarily *c*-type cytochromes, enabling electron transfer from the inner to outer cell membranes *via* electron hopping (Shi et al., 2007; Castelle et al., 2008). Additionally, *G. sulfurreducens* and *S. oneidensis* have been reported to produce conductive pili and outer membrane extensions, respectively, facilitating electron transfer to distantly located solid materials (Gorby et al., 2006; Pirbadian et al., 2014).

In contrast, mediated EET relies on extracellular redox-active low-molecular compounds, referred to as redox mediators, facilitating electron transfer between microbial cells and the environment (Kato, 2015). These redox mediators, whether reduced or oxidized by microorganisms, diffuse to solid surfaces, such as minerals or electrodes, where they either donate or accept electrons. Subsequently, oxidized or reduced mediators return to the cells or enzymes, where they are reused as respiratory substrates. Investigations into mediated EET have revealed that several microorganisms synthesize and excrete such electron mediators, including phenazine and flavin derivatives (Rabaey et al., 2004; Marsili et al., 2008). Additionally, synthetic mediators have found application in microbial fuel cells (Watanabe et al., 2009; Gemünde et al., 2022) and in microbial electrosynthesis (Fruehauf et al., 2020). However, the utilization of mediators often faces substantial challenges in regard to their toxicity and long-term stability (Gemünde et al., 2022).

Microorganisms with direct or mediated EET capabilities have garnered significant attention for their various biotechnological applications, including bioremediation of pollutants (Lovley et al., 2011), metal recovery (Lloyd et al., 1998; Konishi et al., 2007), wastewater treatment (Rodríguez Arredondo et al., 2015; Galeano et al., 2023), energy generation in microbial fuel cells (Obileke et al., 2021; Nawaz et al., 2022), electrochemical modulation of microbial metabolism (Flynn et al., 2010; Lu et al., 2014), and stimulation of microbial symbiotic reactions (Summers et al., 2010; Kato et al., 2013), alongside microbial or enzymatic electrosynthesis (Chen et al., 2020; Wu et al., 2023). One such microorganism of particular interest is *Dehalococcoides mccartyi* strain CBDB1, a strictly anaerobic bacterium, which gained prominence for its ability to reductively dehalogenate a variety of toxic and persistent halogenated organic compounds, such as halogenated benzenes (Jayachandran et al., 2003; Wagner et al., 2012), ethenes (Marco-Urrea et al., 2011), and phenols (Adrian et al., 2007), among others.

*D. mccartyi* strains use halogenated organics as terminal electron acceptor and hydrogen as a sole electron donor for growth through a process known as organohalide respiration (OHR) (Adrian et al., 2000), and they are widely used for bioremediation of soil and groundwater at contaminated sites (e.g., Terra Systems, Claymont, and Sensatec GmbH, Kiel) (Dugat-Bony et al., 2012). OHR occurs at the membrane-bound OHR protein complex, independent of cytochromes and quinones (Schipp et al., 2013). The OHR complex facilitates electron flow on the outer side of the membrane, with all enzymes' active sites facing the periplasm, thus rendering it accessible for electrodes (Kublik et al., 2016). This complex comprises seven subunits arranged in three functional modules (Seidel et al., 2018): (i) the input module, composed of the [Ni-Fe] hydrogenase subunits HupL and HupS, oxidizes hydrogen and conducts the obtained electrons *via* [4Fe-4S] clusters to the central module; (ii) the central module, composed of HupX, OmeA, and OmeB, conducts electrons to the output module and couples this electron flux to proton translocation; and (iii) the output module consisting of the reductive dehalogenase RdhA, which is anchored to the membrane by RdhB, catalyzes the reduction of halogenated compounds. Our previous work has demonstrated that the reduction of the organohalides by RhdA induces proton translocation across the membrane (Hellmold et al., 2023). This finding led to the hypothesis that transferring electrons from RdhA's active site to an anode could also promote proton translocation and thus enabling growth of *D. mccartyi* strains.

For *D. mccartyi* strains, coupling to a cathode has been observed in previous studies, where bioelectrochemically assisted reductive dehalogenation was facilitated using both hydrogen as a natural cathodic mediator (Aulenta et al., 2008) and methyl viologen as a synthetic one (Aulenta et al., 2007, 2010). The advantage of such cathodic OHR over traditional methods is that the microorganisms receive electrons directly from a cathode, eliminating the need for hydrogen as an electron donor. Thus, coupling of organohalide respiring bacteria to a cathode holds great promise for enhancing the reliability, sustainability, and public acceptance of *in-situ* bioremediation of contaminated sites (Adrian and Löffler, 2016).

**Figure 1:**
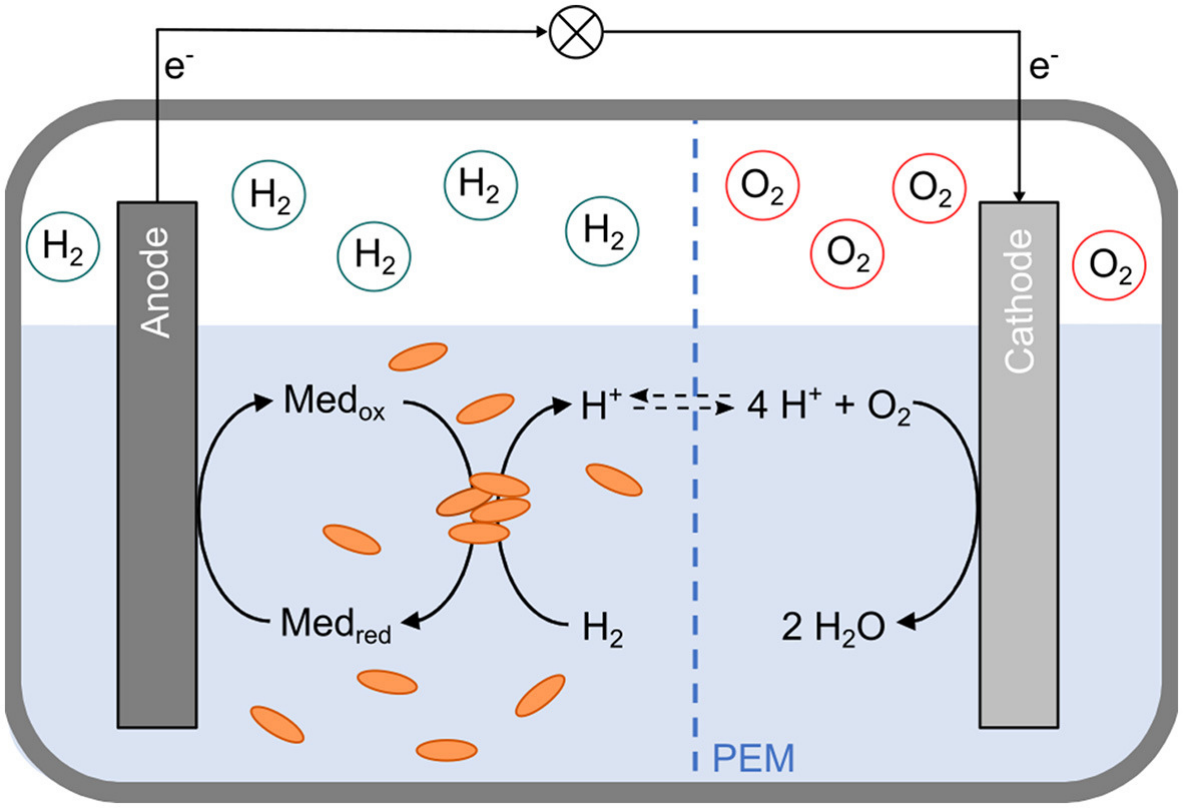
Schematic representation of a two-chamber bioelectrochemical cell for anodic cultivation of *Dehalococcoides mccartyi* strain CBDB1. The cell consists of anodic and cathodic half-cells, separated by a proton exchange membrane (PEM, blue dashed line). The headspace of the anodic compartment contains hydrogen (H_2_), while the cathodic compartment contains oxygen (O_2_). In the anodic half-cell, *D. mccartyi* cells (orange ovals) facilitate the oxidation of hydrogen *via* the [Ni-Fe] hydrogenase HupL of the organohalide respiratory (OHR) protein complex. The electrons are transferred through the OHR complex and finally to an anode mediator (Med_ox_). The reduced mediator (Med_red_) is re-oxidized at the anode. Electrons (e^−^) flow from the anode to the cathode through an external electric circuit. In the cathodic half-cell, these electrons are used to reduce oxygen, forming water (H_2_O).

However, neither direct nor mediated EET between *D. mccartyi* and an anode has been described yet. We hypothesized that employing a mediator capable of reversibly shuttling electrons between RdhA and the anode could enable anodic growth and proliferation of *D. mccartyi* cells without using toxic halogenated compounds. These cells could subsequently serve as an inoculum for *in-situ* bioremediation sites. In a two-chamber microbial fuel cell, hydrogen could serve as an electron donor for *D. mccartyi*, and a specific anode mediator could shuttle electrons between cobalamin in RdhA's active site and the anode. After the reduced anode mediator is re-oxidized, electrons would move to the cathode chamber, where a reduction reaction, such as the conversion of oxygen to water, could occur (Figure 1).

The objective of this study was to identify putative anode mediators capable of being specifically reduced by the OHR complex of strain CBDB1 and shuttling electrons between RdhA and the anode. To achieve this, we conducted methyl viologen-based *in-vitro* activity assays and tested over 20 putative anode mediators from different classes. Our investigation revealed that various cobalt-dependent compounds, referred to as cobalt chelates, including cyanocob(III)alamin (CNCbl^III^), methyl cob(III)alamin (MeCbl^III^), and methyl cobaloxime(III) (MeCoOx^III^), were particularly promising candidates. These cobalt chelates were specifically reduced by strain CBDB1, mostly by RdhA, indicating their potential usefulness as anode mediators. Additionally, we evaluated the ability of cobalt chelates to exchange electrons with an electrode using cyclic voltammetry. Our results show that cobalt chelates exchange electrons with both indium tin oxide (ITO) and gold electrodes. Notably, gold electrodes demonstrated reversible electron exchange, particularly with MeCbl^III^, and a significantly higher current flow, approximately 15–20-fold greater than that of ITO electrodes, highlighting their superior suitability as electrode materials for use with potential anode mediators. Furthermore, leveraging an *in-silico*-predicted RdhA structure and molecular docking techniques, we predicted the binding of anode mediators within the active site of RdhA. Altogether, our data demonstrate how *D. mccartyi* strains can be electrically connected to an anode in an electrochemical cell, a topic that will be addressed in follow-up studies.

## 2 Materials and methods

### 2.1 Cultivation, cell counting, cell harvesting and washing

*D. mccartyi* strain CBDB1 cells were cultivated in a synthetic defined mineral medium following established protocols (Adrian et al., 2000; Jayachandran et al., 2003). The medium contained 1 mM 3,5-dibromo-*L*-tyrosine (DBT) as the terminal electron acceptor and 2 mM *L*-cysteine as the reducing agent. Cultures were incubated at 30°C under strictly anoxic conditions, with the headspace purged with nitrogen and supplemented with 0.2 bar hydrogen as an electron donor. When samples were taken, cells were first filtered through a 1.2 μm cellulose acetate syringe filter prior to harvesting to remove precipitates and particles from the medium. Cells were harvested from the filtrate by centrifugation under anoxic conditions at 9,000 rpm and 16°C for 1 h in 50 mL Falcon tubes. Approximately 10% of the filtrate volume was retained as the “cell pellet.” For further enrichment, “cell pellets” were combined and centrifuged again under the same conditions. To subsequently remove the dissolved substrate (DBT) and the reducing agent (*L*-cysteine) from the cells, the “cell pellet” was washed twice by resuspending it in 100 mM sodium phosphate buffer (pH 6.5) and centrifuging again following the same conditions, and retaining around 10% of the liquid. Finally, the “cell pellet” was filtered again through a 1.2 μm cellulose acetate syringe filter. Cell numbers were determined on agarose-coated slides using epifluorescence microscopy, as described previously (Adrian et al., 2007).

### 2.2 Dehalogenase activity assay using reduced methyl viologen as electron donor

To identify anode mediators, which are reduced by RdhA, a methyl viologen-based activity assay was conducted under strictly anoxic conditions, as previously described (Hölscher et al., 2003). In brief, enzyme activity assays were prepared in 100 mM potassium acetate buffer (pH 5.8), containing 1 mM methyl viologen as an artificial electron donor. The methyl viologen (MV^2+^) was partially reduced by 0.5 mM titanium(III)citrate to generate the highly active radical form (MV^•+^), as the fully reduced form (MV^0^) is inactive in the dehalogenase assay.

The following experimental setups were prepared in triplicate:

(1) Positive control (PC) with 0.3 mM 2,4,6-tribromophenol (TBP) as the electron acceptor.

(2) No-cell-control (NCC) contained an equal volume of 100 mM sodium phosphate buffer (pH 6.5) in place of cells.

(3) No-substrate-control (NSC) contained an equal volume of the mediator or substrate solvent (acetone) instead of the electron acceptor.

More than 20 putative anode mediators at a concentration of 0.3 mM, replacing TBP in the assays, spanning different chemical classes such as (i) dyes that are described to shuttle electrons at different redox potentials, (ii) quinones that typically shuttle electrons between different respiration proteins within the cytosolic membrane, (iii) halogenated compounds that could interact with the RdhA in a similar mechanism as the natural substrate, but which would take up the electrons without being reductively dehalogenated, and (iv) metal chelates that might directly interact with the cobalt atom of the RdhA cofactor (Supplementary Table 1), were tested. The mediator methyl cobaloxime(III) (MeCoOx^III^) was provided by Prof. Dr. Carola Schulzke (University of Greifswald, Germany). Other mediators were purchased in high purity (>95%) from various suppliers, including Merck. Each experiment was reproduced at least three times.

The activity was monitored photometrically at 630 nm and 30°C for 12 h, with absorbance measurements taken every 5 min. Subsequently, the dehalogenase enzyme activity was calculated from the initial decreasing absorbance slope of reduced methyl viologen at 630 nm, using an extinction coefficient of ε_630_ = 11,000 M^−1^ cm^−1^, corrected by subtracting the absorbance changes of NSC and NCC. Since all activity assays were conducted with intact *D. mccartyi* strain CBDB1 cells and not with crude extracts or purified proteins, enzyme activities were normalized to one cell (Equation 1) not to a protein content. This normalization allowed for the calculation of turnover described as the total number of electron acceptor converted per second and cell (s^−1^ cell^−1^).


(1) $$\begin{array}{l} Turnover\;\left(s^{-1}{\;cell}^{-1}\right)\;=\frac{\left(\Delta E_{Sample}\;-\;\Delta E_{NSC}\;-\;\Delta E_{NCC}\;\right)\times V_{Total}\;\times N_{A}}{\varepsilon_{630}\times \;V_{Sample}\;\times \;n_{Cells}\;\times \;d\;\times \;n} \end{array}$$

Δ*E* – change of absorbance (s^−1^) in the sample, NSC (no-substrate-control) or NCC (no-cell-control)

*V*_*Total*_ – total volume per assay (220 μL)

*N*_*A*_ – Avogadro constant (6.02 × 10^20^ mmol^−1^)

ε_630_ – extinction coefficient of methyl viologen (11,000 M^−1^ cm^−1^)

*V*_*Sample*_ – cells volume in the assay (20 μL)

*n*_*Cells*_ – applied cell number (cells mL^−1^)

*d* – optical path length (1 cm)

n – molecules of methyl viologen needed to reduce one molecule of electron acceptor (2).

### 2.3 Dehalogenase activity assay using hydrogen as electron donor

To explore the native dehalogenase activity, we conducted activity assays with hydrogen as an electron donor, while using a halogenated substrate (DBT) as the positive control or an anode mediator as electron acceptor. The tested anode mediators included cyanocob(III)alamin (CNCbl^III^), methyl cob(III)alamin (MeCbl^III^), and methyl cobaloxime(III) (MeCoOx^III^). All assays were prepared within a Coy anaerobic chamber using approximately 4% (v/v) hydrogen in the chamber's atmosphere as the electron donor. The activity assays were set up in 100 mM sodium phosphate buffer (pH 6.5) in GC crimp vials, with a total volume of 2 mL containing 0.4 mL of *D. mccartyi* “cell pellet” and 0.3 mM of the halogenated electron acceptor DBT (positive control) or 0.1 mM of the mediators CNCbl^III^, MeCbl^III^, or MeCoOx^III^ (experimental setups). Additionally, several controls were prepared: (i) a NCC containing 0.4 mL sodium phosphate buffer (pH 6.5) instead of cells with a hydrogen-containing headspace, and (ii) a negative control (NC) where the hydrogen-containing headspace was replaced by nitrogen. NCCs and NCs were prepared in triplicates, while experimental samples were set up in five replicates. Due to the slower native dehalogenase activity with hydrogen as the electron donor compared to the methyl viologen-based *in-vitro* activity (Jayachandran et al., 2004; Hartwig et al., 2017), samples were incubated at room temperature in the dark within the chamber for several days. Subsequently, the concentration of DBT in the positive control vials was quantified using HPLC, as described in the subsequent section, while the reduction of mediators was monitored by recording absorbance spectra between 250 and 700 nm under anoxic conditions within the anaerobic chamber.

### 2.4 Abiotic reduction of cobalt chelates using cob(I)alamin

To investigate whether the reduced corrinoid cofactor of RdhA from strain CBDB1 can reduce the assessed cobalt chelates, we used cob(I)alamin (Cob^I^) to mimic the Co^1+^ state of the corrinoid cofactor in RdhA's active site. We first reduced hydroxocob(III)alamin (OHCbl^III^) by incubating it with zinc powder overnight under anoxic conditions. After confirming Cob^I^ formation, recognizable by an absorbance maximum at 390 nm, the zinc powder was removed by centrifugation at 14,500 × g for 5 min. The supernatant containing Cob^I^ was mixed with equimolar amounts (100 μM each) of MeCbl^III^ or MeCoOx^III^. In parallel, controls using OHCbl^III^ instead of Cob^I^ mixed with the mediators were prepared. Subsequently, UV-Vis absorbance spectra were recorded at the onset, and after 30 min, 1 h, and 3 h of incubation to track the changes. The re-oxidation of Cob^I^ to cob(II)alamin (Cob^II^) was observed through a decrease in absorbance at 390 nm and an increase at 474 nm. In parallel, the formation of reduced mediators (MeCbl^II^ or MeCoOx^II^) was observed through an increase in absorbance at 474 nm and 438 nm, respectively.

### 2.5 HPLC analysis of halogenated substrates and mediators

The halogenated electron acceptor DBT used as a substrate for positive controls, was analyzed *via* HPLC using an UltiMate 3000 System (Thermo Fisher Scientific). The system was equipped with an LiChrospher C_18_ column (125 mm × 4 mm × 5 μm; Merck KGaA Darmstadt/Germany). Solutions containing 0.1% (v/v) formic acid in water (HPLC grade) and 100% methanol were used as eluent A and eluent B, respectively. For the separation of DBT and its debrominated products, a 25-min gradient was used at 20°C with a flow rate of 0.2 mL min^−1^. The gradient started at 30% (v/v) eluent B, increased to 95% eluent B (v/v) over 15 min, held for 4 min, and then returned to the starting condition within 1 min. To detect and quantify eluting compounds, the diode array detector (DAD) was set to monitor wavelengths of 233 nm, 254 nm, 360 nm, and 520 nm.

### 2.6 Electrochemical characterization of redox mediators using cyclic voltammetry

To investigate whether the investigated cobalt chelates, which were probably reduced by RdhA, are exchanging electrons with an electrode, cyclic voltammetry was conducted. All experiments were performed under anoxic conditions within an anaerobic chamber at room temperature, in a defined gas atmosphere containing approximately 3.5% hydrogen in nitrogen. Four different materials for the working electrode—gold, indium tin oxide (ITO), glassy carbon, and platinum were tested. For the gold working electrode, manufactured ItalSens Gold SPE preprinted electrodes (35 mm substrate length, blank surface) were used. These electrodes featured a 3.14 mm^2^ surface gold working electrode, a gold counter electrode, and a silver pseudo-reference electrode. For the ITO working electrode, a multi-electrode array (MEA) was utilized, comprising an ITO working electrode with a 2.84 mm^2^ surface, a platinum counter electrode, and an Ag/AgCl/1 M KCl reference electrode. The MEAs were fabricated as previously described (Frank et al., 2018). Before use, ITO electrodes were pre-cleaned by cycling the electrode potential between 0 V and −0.95 V in 1 M HCl at a scan rate of 50 mV s^−1^ for 3 cycles. Afterwards, the electrodes were rinsed with ddH_2_O. Cyclic voltammograms were recorded using a Sensit Smart potentiostat (PalmSens) and PSTrace Software (PalmSens) at scan rates ranging from 25 mV s^−1^ to 1,000 mV s^−1^. Oxidation (anodic) and reduction (cathodic) peaks, characterized by positive or negative currents, were defined according to the IUPAC convention. Redox potentials were calculated as the midpoint potential between oxidation and reduction peaks and subsequently converted to the potential against the SHE. The measuring solutions consisted of 0.3 mM anode mediators (CNCbl^III^, MeCbl^III^, or MeCoOx^III^) in 0.5 M KCl electrolyte solution.

### 2.7 Bioinformatics, protein structure prediction and protein-ligand docking

The data for the protein structure of the reductive dehalogenase *Dh*PceAB (PDB: 8Q4H) from *Desulfitobacterium hafniense* strain TCE1 was downloaded from the Protein Database (PDB) (Berman et al., 2000). The amino acid sequence of the reductive dehalogenase CbrA (locus cbdbA84) from *D. mccartyi* strain CBDB1 was obtained from the National Center for Biotechnology Information (NCBI) Database (Sayers et al., 2022). The N-terminal signal peptide sequence of CbrA was identified using SignalP 6.0 (Ashkenazy et al., 2016), and subsequently removed from the sequence prior to CbrA protein structure prediction using the AlphaFold2 ColabFold platform (Mirdita et al., 2022). Cofactor binding sites for CbrA were determined by the COFACTOR server (Roy et al., 2012; Zhang et al., 2017). The docking area in CbrA of *D. mccartyi* strain CBDB1 was defined according to the active center of *Dh*PceA from *D. hafniense* strain TCE1 (Cimmino et al., 2023). Ligand structures of the mediators MeCoOx^III^ and MeCbl^III^ were obtained from either the PDB or from PubChem databases (Kim et al., 2023), and then converted to pdb format using OpenBabel (O'Boyle et al., 2011). Receptor-ligand docking was performed using UCSF Chimera (version 1.16) (Pettersen et al., 2004). Therefore, the receptor structures were first aligned using Matchmaker. The ligand and receptor structures were then prepared for docking with the DockPrep function, whereby hydrogen and charges were assigned. Gasteiger charges were used for non-standard residues and AMBER ff14SG for standard residues. Subsequently, docking was conducted with AutoDock Vina docking tool (version 1.2.5) (Eberhardt et al., 2021) using default settings. In order to compare the surface area of MeCbl^III^ and MeCoOx^III^ with the halogenated electron acceptors bromophenol blue (BPB) and 2,3,7,8-tetrachlorodibenzo-*p*-dioxin (TCDD) of *D. mccartyi* strain CBDB1, we estimated the size of all four compounds by calculating the inertia ellipse of the molecular atoms simplifying the molecular structure. The inertia measurement function available in ChimeraX (Meng et al., 2023) was applied using default settings.

## 3 Results

The aim of our study was to identify and characterize substances facilitating electron transfer between RdhA of *D. mccartyi* strain CBDB1 and an anode, thereby functioning as anode mediators. To achieve this, we initially used a methyl viologen-based *in-vitro* activity assay to identify substances that can be reduced by strain CBDB1 in a high throughput. With this approach, we tested over 20 chemicals from four different functional classes (Supplementary Table 1). In particular cobalt chelates, were reduced by strain CBDB1. Next, we established a native assay using hydrogen as the electron donor, instead of reduced methyl viologen, and cobalt chelates as the terminal electron acceptors. In the experimental section, we specifically focused on methyl cob(III)alamin (MeCbl^III^) and methyl cobaloxime(III) (MeCoOx^III^). Finally, we investigated whether these compounds could exchange electrons with an electrode using cyclic voltammetry, and conducted docking studies with the *in-silico* structure of RdhA and MeCbl^III^ as well as MeCoOx^III^.

### 3.1 Identification of putative redox mediators using a methyl viologen-based activity assay

**Figure 2:**
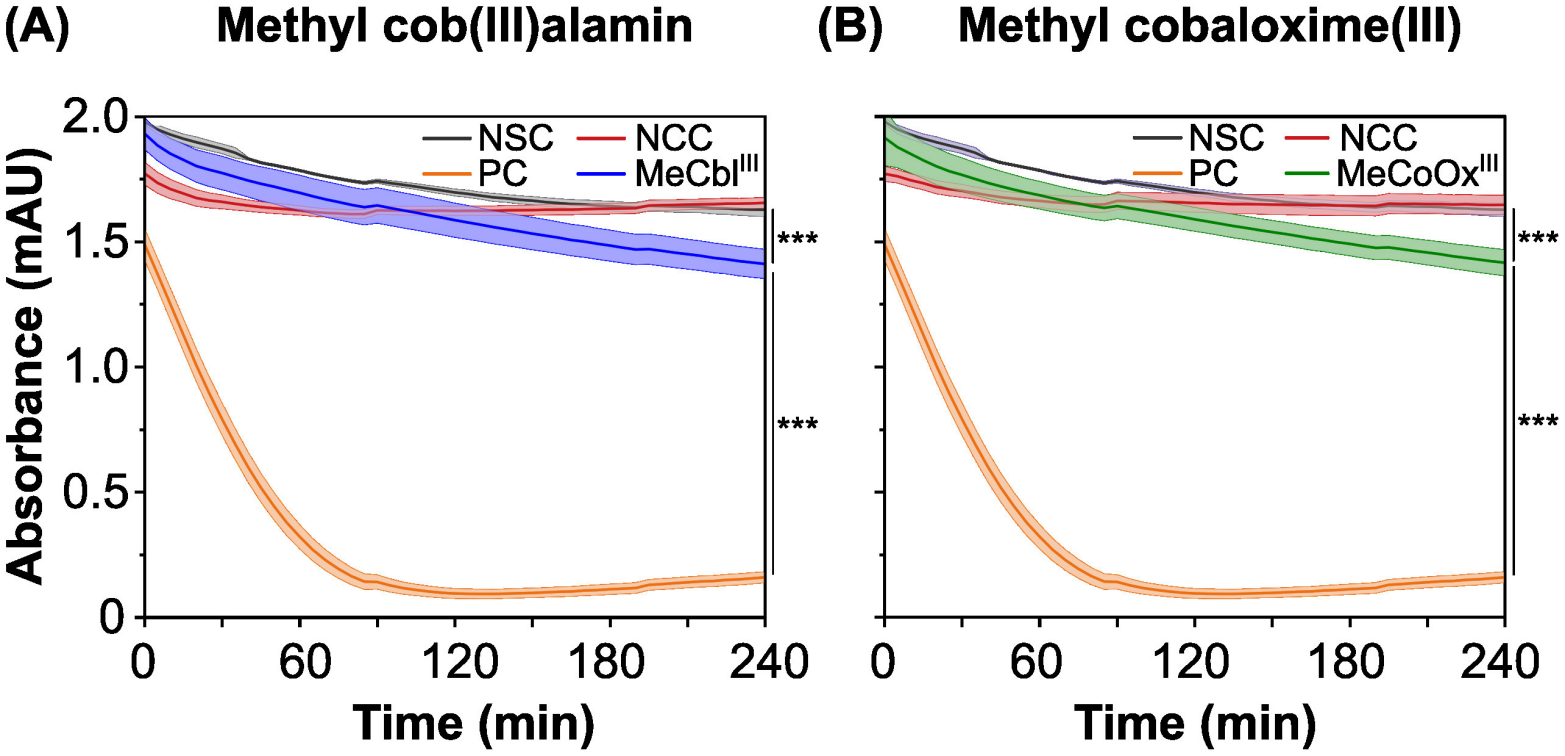
Absorbance at 630 nm over time in photometric *in-vitro* activity assays, using reduced methyl viologen as the electron donor for the reductive dehalogenase (RdhA). The absorbance changes correlate with the re-oxidation and concentration of reduced methyl viologen. The activity assays were performed in five replicates, with the mean represented by a line and the standard deviation by the filled area under the line. **(A)** Methyl viologen oxidation in the presence of methyl cob(III)alamin (MeCbl^III^, blue line). A positive control (PC, orange line) with 2,4,6-tribromophenol (TBP), a no-cell-control (NCC, red line) containing MeCbl^III^ and a no-substrate-control (NSC, gray line) containing *D. mccartyi* strain CBDB1 cells were included. **(B)** Methyl viologen oxidation in the presence of methyl cobaloxime(III) (MeCoOx^III^, green line). A positive control with TBP (orange line), as well as an NCC (red line) containing MeCoOx^III^ and an NSC were included. Significances were determined using the one-way ANOVA analysis test with ^***^: *p* < 0.001.

To identify suitable mediators for electron transfer between RdhA and an anode, we tested over 20 putative electron acceptors using a photometric high throughput methyl viologen-based activity assay. A positive control (PC) with 2,4,6-tribromophenol (TBP) as the terminal electron acceptor was included, along with a no-substrate-control (without the electron acceptor) and a no-cell-control to track the abiotic re-oxidation of reduced methyl viologen by non-cellular compounds. While the absorbance of the no-cell-control remained stable (Figure 2, red line) indicating that the putative mediators were stable against abiotic reduction by methyl viologen, the absorbance of the no-substrate-control slightly decreased (Figure 2, gray line), likely due to residual substrate (DBT) in the medium that was difficult to completely remove by washing. Most putative mediators were stable against reduction by strain CBDB1, while neutral red was abiotically reduced by methyl viologen in the no-cell-control (Supplementary Table 1). Although the dyes phenol red and indigo blue, as well as the metal complex potassium oxalate iron(III) chloride, were biotically reduced by strain CBDB1, they were also reduced in the NCC when concentrations of reducing agents titanium(III) citrate and methyl viologen were increased (data not shown).

However, our study focused on cobalt chelates, such as MeCbl^III^ and MeCoOx^III^, which were reduced by strain CBDB1 in the methyl viologen-based activity assay (Figure 2, blue and green lines). This was demonstrated by a decrease in the absorbance of reduced methyl viologen at 630 nm, putatively caused by the reduction of the cobalt chelates by RdhA. A significantly higher decrease in absorbance (*p* < 0.001) was observed in positive controls, where TBP was used as the terminal electron acceptor (Figure 2, orange line). Similar observations were also made for cyanocob(III)alamin (CNCbl^III^) (Supplementary Figure 1).

From the absorbance changes at 630 nm, corrected by subtracting the absorbance changes of NSC and NCC, we calculated the turnover rate as the absolute number of halogenated substrates or mediators converted per second per cell (Equation 1). The turnover rates for MeCbl^III^ and MeCoOx^III^ by strain CBDB1 were in a similar range (*p* = 0.47), amounting to 25,000 and 19,000 s^−1^ cell^−1^, respectively, which is significantly lower (*p*-value <0.001) compared to the turnover of the halogenated substrate TBP amounting to 370,000 conversions per second per cell.

### 3.2 Hydrogen-dependent reduction of cobalt chelates

To determine whether cobalt chelates can be reduced by RdhA with hydrogen as electron donor we conducted an activity assay with whole cells of strain CBDB1. In these assays, hydrogen served as the electron donor, while putative mediators or DBT acted as the terminal electron acceptor. The reaction mixtures were incubated for 8 days in an anaerobic chamber in the dark. Similarly to the methyl viologen-based assay, a positive control with DBT as the terminal electron acceptor as well as a no-cell-control were included. Additionally, a negative control, where hydrogen in the headspace was replaced with nitrogen (removing the electron donor), was added.

**Figure 3:**
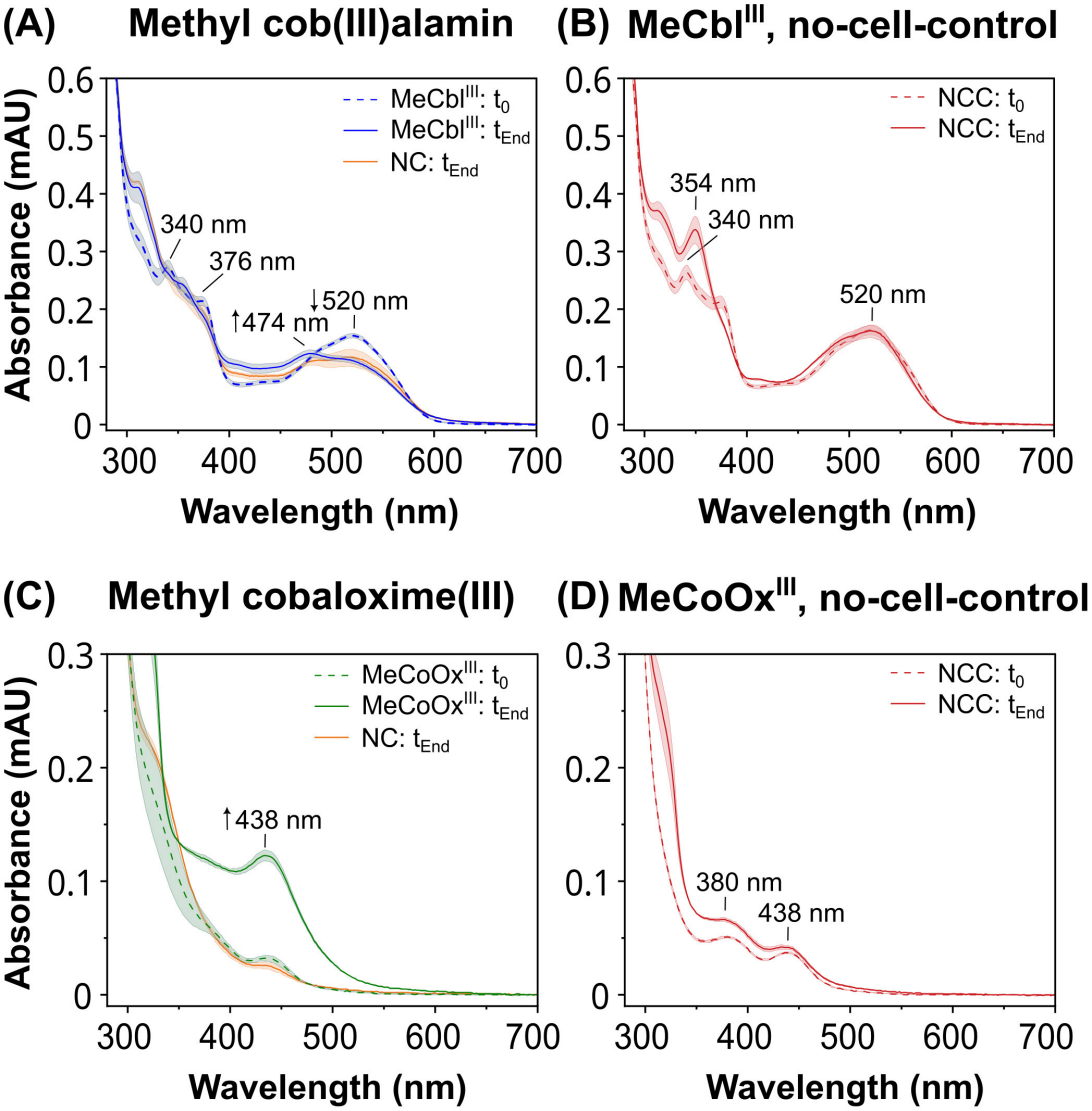
UV-Vis absorbance spectra of an activity assay using hydrogen as the electron donor and anode mediators methyl cob(III)alamin (MeCbl^III^) or methyl cobaloxime(III) (MeCoOx^III^) as the electron acceptors. Spectra were recorded at the beginning (t_0_) of the reaction and after 8 days (t_End_). Samples containing putative anode mediators were set up in five replicates, controls in triplicates. Means are represented by the lines, and standard deviations are shown as filled areas under the lines. In the negative controls (NC) where the mediators were present but the hydrogen-containing headspace was replaced with nitrogen, only the UV-Vis spectra at t_End_ are shown (orange lines). **(A)** Absorbance spectra of samples containing MeCbl^III^ (blue lines) and the NC with nitrogen in the head space (orange line) shown at t_0_ (dashed line) and at t_End_ (continuous line) of the incubation. **(C)** Absorbance spectra of samples containing MeCoOx^III^ (green lines) and the NC (orange line) at t_0_ (dashed line) and at t_End_ (continuous line). Absorbance spectra of no-cell-controls (NCC) in hydrogen head space containing **(B)** MeCbl^III^ or **(D)** MeCoOx^III^ as electron acceptors at t_0_ (dashed line) and t_End_ (continuous line).

Assessment of the hydrogen-dependent reductive dehalogenation of DBT in the positive control by quantifying DBT and its debromination products by HPLC showed that DBT was completely debrominated after 24 h of incubation (Supplementary Figure 2). In contrast, the reduction of cobalt chelates by strain CBDB1 was monitored by recording UV-Vis spectra (Figure 3). At the onset of the incubation, MeCbl^III^ exhibited pronounced absorbance peaks at 340 nm, 376 nm and 520 nm in all setups (Figure 3A), which are typical for MeCbl^III^ (Toda et al., 2019). In the setup containing CBDB1 cells and the MeCbl^III^ mediator, the peaks at 340 nm and 520 nm decreased with the progression of incubation (Figure 3A, blue line), and a new distinct peak at 474 nm appeared, which is specific for Cob^II^ (Toda et al., 2019). The concentration of Cob^II^ formed after incubation was determined to be approximately 60 μM, using its extinction coefficient at 474 nm (ε_474_ = 8,200 M^−1^ cm^−1^). However, the absorbance spectrum of the negative control also changed, showing a decrease in absorbance at 520 nm and disappearance of the 340 nm and 376 nm peaks, but without the formation of an absorbance maximum at 474 nm (indicative for Cob^II^). These changes might be caused by the uptake or reactivity of the CBDB1 cells with MeCbl^III^. In the no-cell-control, the absorbance peak for MeCbl^III^ at 340 nm shifted to 354 nm, indicating the interconversion of MeCbl^III^ to OHCbl^III^ (Juzeniene and Nizauskaite, 2013; Toda et al., 2019), while the absorbance at 520 nm remained unchanged (Figure 3B).

The MeCoOx^III^ mediator exhibited two faint peaks at 380 nm and 438 nm in all setups at the onset of the reaction (Figures 3C, D). In setups containing MeCoOx^III^ and CBDB1 cells, the absorbance peak at 380 nm disappeared, while the peak at 438 nm (indicative for MeCoOx^II^) increased more than 3-fold compared to the start of the experiment (Figure 3C). The concentration of MeCoOx^II^ formed after incubation was determined to be approximately 109 μM, based on its extinction coefficient at 438 nm (ε_438_ = 4,400 M^−1^ cm^−1^). No significant increase in the absorbance at 438 nm was observed in the no-cell-control, where CBDB1 cells were replaced with buffer (Figure 3D), or in the negative control, where hydrogen in the headspace was replaced with nitrogen (Figure 3C). Although the absorbance at 380 nm has also disappeared in the negative control, the peak at 438 nm did not increase.

In conclusion, our data indicate that strain CBDB1 is capable of hydrogen-dependent reduction of MeCbl^III^ to Cob^II^ and MeCoOx^III^ to MeCoOx^II^. Furthermore, our activity assay data, using hydrogen as the electron donor, indicate that the reduction of MeCbl^III^ to MeCbl^II^ by strain CBDB1 is more strongly limited compared to MeCoOx^III^, as less Cob^II^ was formed in comparison to MeCoOx^II^.

### 3.3 Abiotic reduction of cobalt chelates by cob(I)alamin

**Figure 4:**
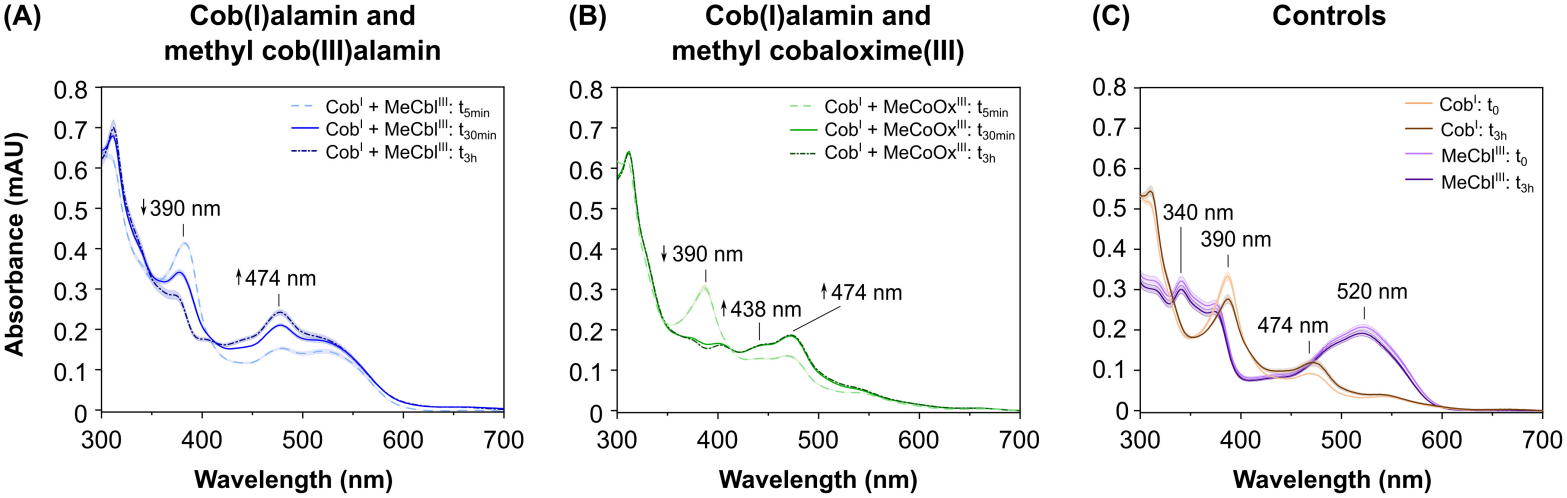
UV-Vis absorbance spectra of the abiotic reduction of the anode mediators methyl cob(III)alamin (MeCbl^III^) and methyl cobaloxime(III) (MeCoOx^III^) using cob(I)alamin (Cob^I^) as electron donor. Spectra were recorded at the onset of the reaction (t_0_), after 5 min (t_5min_), after 30 min (t_30min_) and/or after 3 h (t_3h_). Samples containing anode mediators were set up in five replicates, controls without mediators in triplicates. **(A)** Absorbance spectra in assays containing Cob^I^ plus mediator MeCbl^III^ (blue lines) at t_5min_, at t_30min_, and t_3h_. **(B)** Absorbance spectra in assays with Cob^I^ plus mediator MeCoOx^III^ (green lines) at t_5min_, t_30min_, and t_3h_. **(C)** Absorbance spectra of control assays containing Cob^I^ alone (brown lines) or MeCbl^III^ alone (violet lines) at t_0_ and t_3h_.

To investigate whether RdhA from strain CBDB1 is involved into the reduction of cobalt chelates, we used cob(I)alamin in abiotic experiments to mimic the Co^1+^ corrinoid cofactor of RdhA. In these experiments, we mixed cob(I)alamin with MeCbl^III^ or MeCoOx^III^ in an equimolar ratio. The UV-Vis absorbance spectra were recorded at the onset (5 min after start), as well as after 30 min, 1 h, and 3 h of incubation (Figure 4).

Our data demonstrate that MeCbl^III^ is reduced to MeCbl^II^ by cob(I)alamin. This reduction is evidenced by a decrease in absorbance at 390 nm (indicative of consumption of approximately 22 μM cob(I)alamin) along with an increase in absorbance at 474 nm (indicative of formation of approximately 44 μM cob(II)alamin). The increase at 474 nm originates from both the production of cob(II)alamin by cob(I)alamin oxidation, and the production of MeCbl^II^ by MeCbl^III^ reduction (Figure 4A). In contrast, when not reduced cob(III)alamin in the form of OHCbl^III^ was incubated with MeCbl^III^ (anode mediator), no changes in the absorbance spectrum were observed (Supplementary Figure 3A), indicating no interaction between OHCbl^III^ and MeCbl^III^.

Furthermore, our data show that MeCoOx^III^ is reduced to MeCoOx^II^ by cob(I)alamin, as evidenced by the decrease in absorbance at 390 nm (indicative of consumption of approximately 22 μM cob(I)alamin) and the increase at 438 nm (indicative of formation of approximately 27 μM MeCoOx^II^). Concurrently, the oxidation of cob(I)alamin to cob(II)alamin results in an increase in absorbance at 474 nm amounting to approximately 24 μM Cob^II^ (Figure 4B). In contrast, when OHCbl^III^ was incubated with MeCoOx^III^, absorbance spectrum did not change (Supplementary Figure 3B), indicating that there is no interaction between OHCbl^III^ and MeCoOx^III^.

### 3.4 Electrochemical characterization of cobalt chelates using cyclic voltammetry

**Figure 5:**
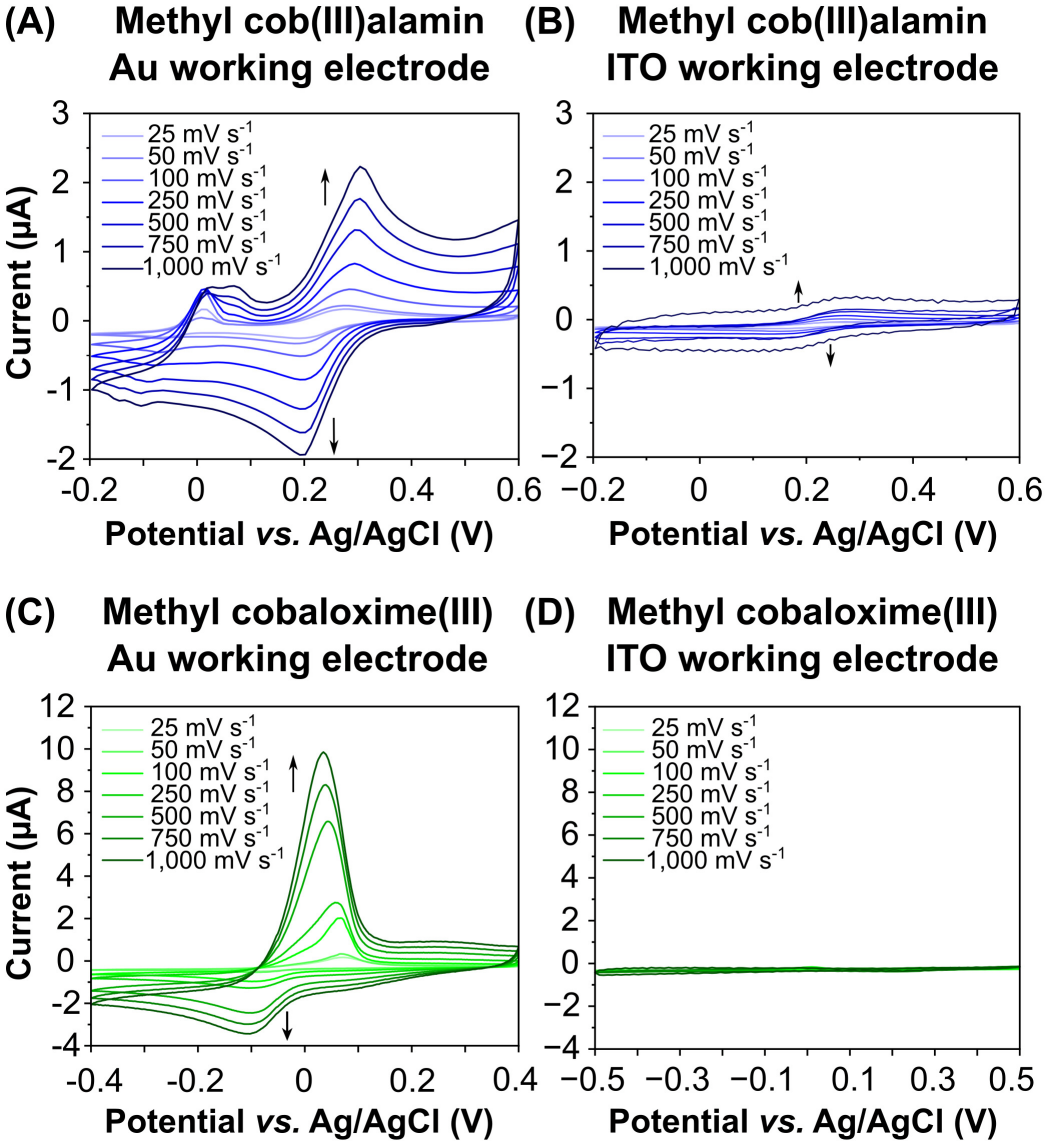
Cyclic voltammograms of the putative anode mediators methyl cob(III)alamin **(A, B)** and methyl cobaloxime (III) **(C, D)** recorded on different working electrode materials and scan rates. Gold (Au) or Platinum was used as a counter electrode and Ag/AgCl as a reference electrode. We compared Au **(A, C)** and indium tin oxide (ITO) **(B, D)** as working electrode materials. Data were recorded at scan rates ranging from 25 to 1,000 mV s^−1^. The peaks at more positive potentials represent the oxidation of Co(II) to Co(III), while the peaks at the more negative potentials indicate the reduction of Co(III) to Co(II).

To determine whether the cobalt chelates can exchange electrons with an electrode we employed cyclic voltammetry, testing four working electrode materials: gold, ITO, glassy carbon, and platinum. No interaction between cobalt chelates and the glassy carbon or platinum working electrodes was observed (data not shown), indicating that these electrodes are unsuitable for our system. Exemplary, the cyclic voltammograms for MeCbl^III^ and MeCoOx^III^ with gold (Figures 5A, C) and ITO (Figures 5B, D) working electrodes are shown.

Prominent oxidative and reductive peaks were observed for both MeCbl^III^ and MeCoOx^III^ when gold was used as the working electrode. Oxidation peaks indicated the conversion of Co(II) to Co(III), while reduction peaks showed the reverse reaction. For both putative anode mediators, the determined redox potentials *vs*. SHE were positive. The redox potential of MeCbl^III^ on a gold working electrode was +240 ± 10 mV (+440 ± 10 mV *vs*. SHE), while on an ITO working electrode it was 200 ± 10 mV (+400 ± 10 mV *vs*. SHE). A second oxidation peak for MeCbl^III^ was observed with increasing scan rates on the gold working electrode (Figure 5A), but no corresponding reduction peak was detected, even when the potential range was expanded. The current flow on the gold working electrode was 4–12 times higher than on the ITO working electrode, depending on the scan rates (Figure 5B).

The redox potential of MeCoOx^III^ on a gold working electrode was −25 ± 15 mV (+175 mV ± 15 mV *vs*. SHE) (Figure 5C), and −165 ± 25 mV (+35 ± 25 mV *vs*. SHE) on an ITO working electrode (Figure 5D). The current flow for MeCoOx^III^ on the gold working electrode was 21–55 times higher than on the ITO working electrode (Figures 5C, D). Additionally, the oxidation peak of MeCoOx^III^ was 2–3 times higher than its corresponding reduction peak, indicating irreversible electron transfer and that MeCoOx^III^ favors oxidation at the gold electrode (Figure 5C).

To determine whether MeCbl^III^ and MeCoOx^III^ reversibly exchange electrons with the gold or ITO working electrodes, we calculated the peak potential separation (Δ*E*_p_) between the oxidation (*E*_pa_) and reduction (*E*_pc_) peaks, as well as the peak current ratio (*i*_pc_/*i*_pa_) (Supplementary Table 2). For MeCbl^III^, Δ*E*_p_ was approximately 93 ± 8 mV or 111 ± 11 mV, and *i*_pc_/*i*_pa_ was 0.84 ± 0.05 or 0.82 ± 0.12 for the gold and ITO electrodes, respectively, indicating a reversible electron transfer. In contrast, Δ*E*_p_ for MeCoOx^III^ was significantly higher, measuring 146 ± 8 mV for the gold electrode and 251 ± 107 mV for the ITO electrode. The large Δ*E*_p_ for MeCoOx^III^ suggests either irreversible (slow) electron transfer or electron transfer followed by a chemical reaction, altering the product into another species. This irreversible electron transfer for MeCoOx^III^ is further supported by the *i*_pc_/*i*_pa_ values, which were 0.33 ± 0.21 for the gold electrode and 0.51 ± 0.14 for the ITO electrode.

Exemplary current densities were calculated for scan rates of 25 and 1,000 mV s^−1^ for both mediators (Supplementary Table 3). For the oxidation of MeCbl^III^ on gold, we measured current densities of 7 μA cm^−2^ and 63 μA cm^−2^ for the lowest and highest scan rates, respectively. On ITO, the current densities were up to 10 times lower, amounting to 2 μA cm^−2^ or 6 μA cm^−2^. The oxidation of MeCoOx^III^ on the gold working electrode resulted in current densities of 19 μA cm^−2^ at 25 mV s^−1^ and 324 μA cm^−2^ at 1,000 mV s^−1^. On ITO, the current densities were lower, amounting to 1 μA cm^−2^ and 3 μA cm^−2^, respectively.

Taken together, our data indicate that both putative anode mediators, MeCbl^III^ and MeCoOx^III^, are capable to efficiently exchange electrons, particularly with a gold electrode. This suggests that gold could be used as an anode material in future electrochemical reactor setups.

### 3.5 Molecular docking of cobalt chelates to RdhA

**Figure 6:**
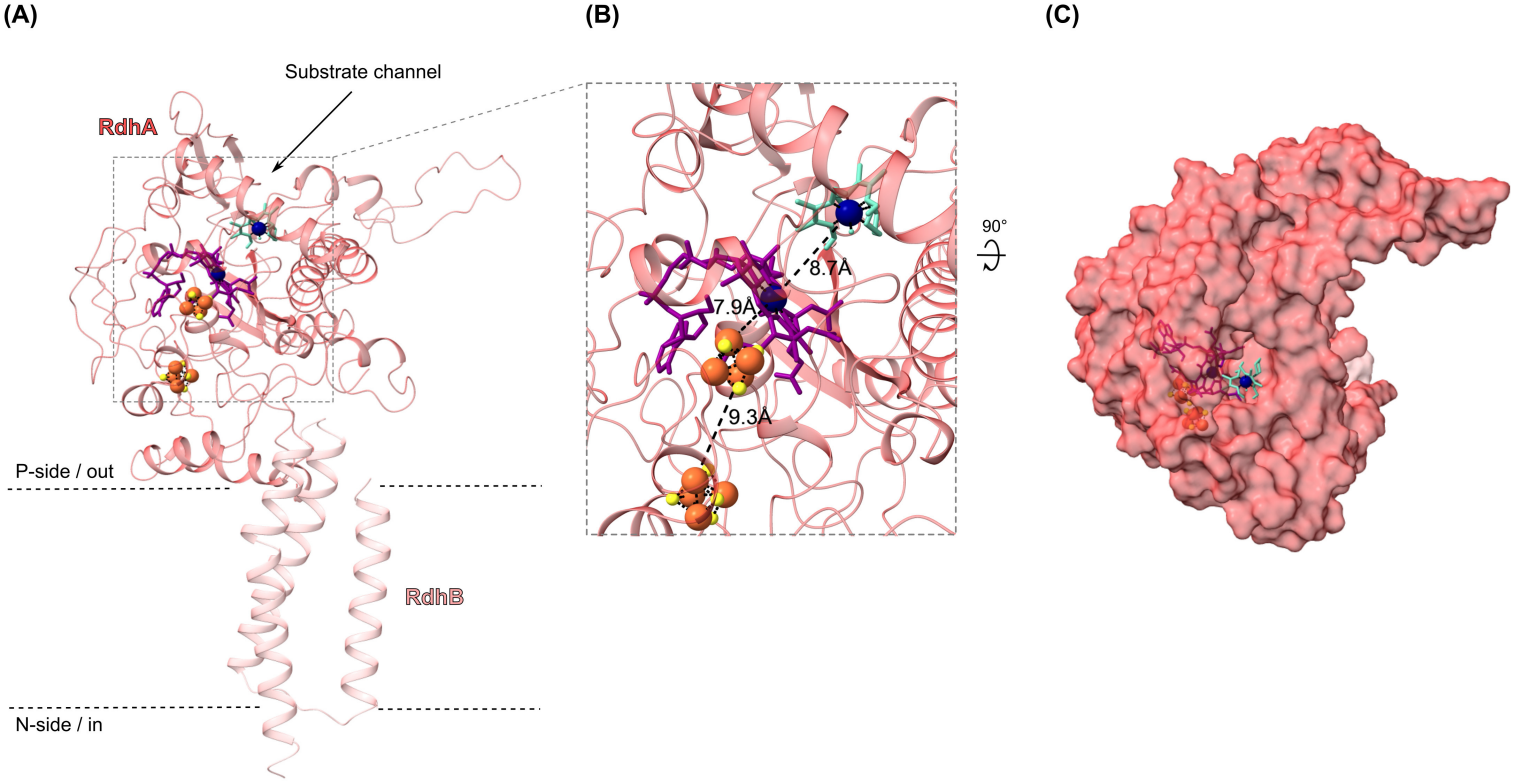
*In-silico* structure of the reductive dehalogenase RdhA (locus cbdbA84) and its anchoring protein RdhB (cbdbA85) from *Dehalococcoides mccartyi* strain CBDB1, predicted with AlphaFold2, including docking of the putative anode mediator methyl cobaloxime(III) (MeCoOx^III^, turquoise) at the enzyme's active center. **(A)** Cartoon representation of RdhA (dark pink) and RdhB (light pink), computed with AlphaFold2 ColabFold platform, embedded into the cytosolic membrane. The binding of the [4Fe-4S] clusters (yellow-orange spheres) and the corrinoid cofactor (purple stick conformation) was defined based on the active center of the *Dh*PceA subunit from *Desulfitobacterium hafniense* strain TCE1. Docking of MeCoOx^III^ (turquoise stick conformation) was performed using AutoDock Vina. Cobalt ions of the corrinoid cofactor and MeCoOx^III^ are shown as dark blue spheres. **(B)** Zoom-in of the active center of RdhA with MeCoOx^III^ docked to the corrinoid cofactor. The distances between the metallocofactors and the putative anode mediator are shown in Angstroms (Å). **(C)** Surface structure of the RdhAB module of strain CBDB1, rotated 90° to the front around the x-axis, showing the substrate channel in RdhA from the top perspective.

To obtain further evidence on a potential binding of cobalt chelates to the corrinoid cofactor in the active site of RdhA to transfer electrons, we conducted molecular docking studies. We selected the OHR submodule composed of the reductive dehalogenase RdhA/CbrA (CbdbA84) and its anchor protein RdhB (CbdbA85) to exemplary represent the 32 RdhAB modules encoded in strain CBDB1. The structure of the RdhAB module was predicted using AlphaFold2, resulting in an overall confidence score of 89.0. In this structure, RdhB is positioned at a right angle below RdhA (Figure 6A). The *in-silico* structure of the RdhA subunit reveals that the active center of the enzyme is accessible from the protein's surface through a substrate tunnel, with the cobalt ion (Co) of the corrinoid cofactor situated at the center of the putative substrate binding channel (Figure 6A). Two [4Fe-4S] clusters, forming a “conductive wire” on the opposite side of the active center, facilitate electron transfer to the corrinoid cofactor from other OHR subunits (Figure 6B). Subsequently, the *in-silico* structure and cofactor positions in the RdhAB module of strain CBDB1 were compared to the *Dh*Pce(AB)_2_ heterodimer structure from *Desulfitobacterium hafniense*, previously published (Cimmino et al., 2023). A notable difference between the two structures is the conformation of the substrate channels, which is wider in the RdhA of strain CBDB1 (Figure 6C).

To analyze the binding compatibility between RdhA and cobalt chelates acting as putative mediators, docking experiments with MeCbl^III^ (Supplementary Figure 4) and MeCoOx^III^ (Figure 6) were conducted. Due to the lack of an experimentally solved RdhAB structure with cofactors for strain CBDB1, docking experiments were initially performed using *Dh*PceA from *D. hafniense*, defining the active site of *Dh*PceA as the docking area. Subsequently, docking was also conducted with RdhA from strain CBDB1. Docking of MeCbl^III^ to the active sites of RdhA and *Dh*PceA resulted in highly unfavorable binding, with binding energies exceeding 400 kJ mol^−1^ in both cases (Supplementary Figure 5). In contrast, docking of MeCoOx^III^ with RdhA from strain CBDB1 resulted in favorable binding, with binding energy of −27.6 kJ mol^−1^, with MeCoOx^III^ precisely fitting into the approximately 9–10 Å wide entry of RdhA's substrate tunnel (Figure 6C). However, docking of MeCoOx^III^ with *Dh*PceA led to non-spontaneous binding (146.4 kJ mol^−1^), which can be attributed to the more closed active site conformation of the enzyme compared to RdhA from strain CBDB1 (data not shown). Despite these differences, the position of MeCoOx^III^ in *Dh*PceA and RdhA showed minimal variation in the aligned proteins (shift of 2.1 Å), indicating that the docked MeCoOx^III^ in RdhA was reasonably positioned, even with an *in-silico* structure. The cobalt ion of the docked MeCoOx^III^ mediator was 8.7 Å away from the Co^1+^ of the corrinoid cofactor of RdhA, possibly allowing electron transfer.

## 4 Discussion

In this study, we identified and described suitable anode mediators that can facilitate electron transfer from the RdhA in *D. mccartyi* strain CBDB1 to an anode. While we did not directly demonstrate electron transfer from RdhA to an electrode *via* the anode mediators in this study, we conducted fundamental research focusing on two essential steps necessary for establishing a bioelectrochemical system for *Dehalococcoides* strains: First, we demonstrated that cobalt chelates, such as methyl cob(III)alamin (MeCbl^III^) and methyl cobaloxime(III) (MeCoOx^III^), are putatively reduced by RdhA from the OHR complex of strain CBDB1 when hydrogen is used as the electron donor. Second, using cyclic voltammetry we showed that the same cobalt chelates exchange electrons with gold electrodes, making them suitable candidates for mediating extracellular electron transfer between RdhA and the anode. This will allow us to design a bioelectrochemical cell for anodic cultivation of strain CBDB1, in which the cells would use, for instance, MeCoOx^III^ or other compounds as a redox mediator for anodic respiration.

When hydrogen was used as the electron donor, both MeCbl^III^ and MeCoOx^III^ were reduced to MeCbl^II^ and MeCoOx^II^, respectively, in the presence of CBDB1 cells (Figure 3). In these activity assays, hydrogen served as the sole electron donor, while halogenated compounds or cobalt chelates acted as electron acceptors, with reduction being strictly hydrogen-dependent. Hydrogen-dependent organohalide reduction is known to occur exclusively *via* the membrane-bound OHR complex, which faces the periplasm and contains RdhA and the Hup hydrogenase responsible for hydrogen oxidation. When hydrogen was replaced with nitrogen in our assays, preventing electron generation by Hup hydrogenase, no reduction of cobalt chelates occurred (Figures 3A, C), indicating that cobalt chelate reduction is tightly coupled to hydrogen oxidation, which can only be mediated by Hup hydrogenase. While we cannot entirely rule out the involvement of other OHR subunits or other proteins in cobalt chelate reduction, this is unlikely. Further evidence supporting RdhA's role in cobalt chelate reduction includes: (i) our docking experiments with the *in-silico* RdhA structure, which showed favorable binding of MeCoOx^III^, and (ii) abiotic experiments where Cob^I^ successfully reduced MeCoOx^III^, a reduction that zinc powder (*E*° = −0.76 V *vs*. SHE) could not achieve. Together, these findings suggest that a cobalamin-dependent enzyme, most likely RdhA—the only cobalamin-dependent enzyme facing the periplasm in strain CBDB1—is essential for cobalt chelate reduction.

Moreover, the native activity test also indicated that the cobalt chelates did not exhibit enzymatic toxicity at 100 μM concentrations. Although our methyl viologen-based activity assays showed that strain CBDB1 reduced approximately 20,000 molecules of MeCbl^III^ and MeCoOx^III^ per second per cell, which was significantly lower to the turnover rate of its natural halogenated substrate TBP the reduction of cobalt chelates with hydrogen as the electron donor was much slower. While RdhA's halogenated substrate DBT (300 μM), which was also used for cultivation, was completely dehalogenated within 24 h of incubation with hydrogen (Supplementary Figure 2A), the reduction of MeCbl^III^ and MeCoOx^III^ took several days under the same conditions (Figure 3). In particular, the reduction of MeCbl^III^ by RdhA was strongly limited in comparison to that of MeCoOx^III^ (Figures 3A, C). This observation aligns with our computational docking studies, which showed that while MeCoOx^III^ precisely fits into the approximately 9–10 Å wide entry of RdhA's substrate tunnel, exhibiting a favorable binding (Figure 6), with binding energy of −27.6 kJ mol^−1^, the docking of MeCbl^III^ to RdhA (CbdbA84) resulted in highly unfavorable binding (binding energy exceeding +400 kJ mol^−1^) likely due to the large size of MeCbl^III^ and the sterically inflexible structure of the corrin ring (Supplementary Figure 4). However, the cobalt atoms of RdhA's corrinoid cofactor and of MeCoOx^III^ and MeCbl^III^ are located at distances of 8.7 Å and 7.5 Å, respectively, which is within a reasonable range for efficient and fast electron transfer (Page et al., 1999).

To support our hypothesis that MeCoOx^III^ is more readily reduced by RdhA than MeCbl^III^ due to its better fit in the substrate tunnel, we calculated the ellipsoidal surface area of these compounds and compared them with bromophenol blue (BPB) and tetrachlorodibenzo-*p*-dioxin (TCDD) (Supplementary Table 4), previously identified as electron acceptors for RdhA (Bunge et al., 2003; Liu et al., 2010; Yang et al., 2015). The surface area of TCDD was found to be similar to that of MeCoOx^III^, amounting to 164 and 153 Å^2^, respectively. In contrast, BPB had a significantly larger surface area of around 365 Å^2^ but was still effectively debrominated by RdhA (Yang et al., 2015). However, the surface area of MeCbl^III^ in both the “base-on” and “base-off” conformation exceeds that of BPB by approximately 2-fold, supporting our observation that MeCbl^III^ is reduced much more slowly by RdhA than MeCoOx^III^ (Figure 3). Our data indicate that RdhAs are not highly specific and can probably reduce a wide range of halogenated aromatic compounds as well as cobalt chelates of a certain size fitting into the substrate tunnel. This indicates that the primary function of RdhA in the OHR complex may be to facilitate electron transfer to diverse compounds in the environment, maintaining electron flow and thus sustaining proton motive force generation.

For a fast electron transfer from the corrinoid cofactor of the OHR complex to an anode mediator a favorable redox potential difference is important (Kavanagh and Leech, 2013). To be applicable, the mediator's redox potential must be similar or more positive than that of the corrinoid cofactor in the active center of RdhA (Kräutler et al., 2003). Our cyclic voltammetry data show that the redox potential of the two cobalt chelates were much more positive (+400 mV *vs*. SHE for MeCbl^III^ and +175 mV *vs*. SHE for MeCoOx^III^) than the redox potential of the Co^1+^ state of the corrinoid cofactor within RdhA's active center, with approximately −380 mV *vs*. SHE (Schumacher et al., 1997; Neumann et al., 1998; Payne et al., 2015). Furthermore, our abiotic experiments showed that cob(I)alamin, which mimicked the Co^1+^ state of the corrinoid cofactor, rapidly transferred electrons to both cobalt chelates MeCbl^III^ and MeCoOx^III^ (Figures 4A, B). The electron transfer between cobalt atoms of different cobalt complexes, as observed in our study, has been described as outer-sphere electron transfer in previous studies, also known as self-exchange reactions (Rillema et al., 1972; Wang and Jordan, 1996; Ullman and Nocera, 2013). Generally, outer-sphere electron transfers are slow, and cobalamin and cobaloxime couples exhibit high self-exchange rates ranging between 2.5 × 10^3^ M^−1^ s^−1^ and 1.4 × 10^2^ M^−1^ s^−1^ (Rillema et al., 1972; Wang and Jordan, 1996). In contrast, inner-sphere electron transfers, such as those occurring between two cobalt atoms of two cobalt complexes mediated by halogens, are significantly faster, reaching exchange rates of 10^5^ M^−1^ s^−1^ (Taube, 1984). This mechanism has also been proposed for dehalogenation reactions (Cooper et al., 2015; Zhang et al., 2023). Inner-sphere electron transfer involves electrons being transferred into the antibonding σ orbital of the cobalt atom *via* a halogen atom, as has been described by Taube (1984). Since dehalogenation reactions typically follow this faster inner-sphere electron transfer mechanism, it could explain the significantly faster conversion of halogenated substrates like DBT compared to MeCoOx^III^ when hydrogen is used as the electron donor (Figure 3), despite MeCoOx^III^ likely fitting well into RdhA's active site (Figure 6). While RdhA is specialized in inner-sphere electron transfer during the reductive dehalogenation step, we have explored the outer-sphere electron transfer capabilities of the corrinoid cofactor to facilitate electron transfer to cobalt chelates in this study.

In this study we primarily focused on cobalt chelates as potential anode mediators, which can pose some risks, such as cell toxicity and irreversible electron transfer, particularly observed for MeCoOx^III^. The irreversible electron transfer was characterized by high peak separation values, shifts in oxidative and reductive peaks with increasing scan rates, and peak current ratios (*i*_pc_/*i*_pa_) uneven 1 (Supplementary Table 2). However, our data cannot definitively determine whether this is due to slow electron transfer between MeCoOx^III^ and the electrode or is caused by a chemical reaction following electron transfer, which would alter the product and make MeCoOx^III^ unsuitable for bioelectrochemical reactors. However, as has been shown in our study, dyes like phenol red and indigo blue, as well as the metal complex potassium oxalate iron(III) chloride, were also biotically reduced by strain CBDB1 (Supplementary Table 1), but due to their instability at high concentrations of titanium(III)citrate, they were not further investigated in this study. These compounds remain promising candidates as anode mediators *in-situ* and should be explored in future studies.

Establishing bioelectrochemical reactors in an industrial setting requires current densities that are both high enough to ensure efficient growth and production rates and sustainable for long-term operation. However, the ideal current density varies depending on the specific process and the organisms involved. For economic viability, generally high current densities in the range of mA cm^−2^ are targeted for microbial electrolysis cells (Angenent et al., 2024), microbial fuel cells (Choudhury et al., 2021), and bioelectrosynthesis systems (Prévoteau et al., 2020). The current density of around 320 μA cm^−2^ observed for MeCoOx^III^ on a gold electrode is promising for initial stages but requires optimization for industrial application. Enhancing current densities to reach higher ranges in mA cm^−2^ and reducing costs is crucial for establishing efficient and economically viable bioelectrochemical reactor for the anodic cultivation of *D. mccartyi* strain CBDB1 at an industrial scale. This can be achieved through several strategies: (i) applying a thin layer of gold to cheaper electrodes to reduce the amount of gold needed; (ii) increasing the surface area of low-cost electrodes (e.g., through nanostructuring) before gold coating to enhance current densities, making the electrodes more suitable for industrial applications; (iii) coating inexpensive materials, e.g., graphite, with conductive polymers, nanomaterials, or other modifications to improve electron transfer and narrow the performance gap between costly materials like gold and lower-cost alternatives; and (iv) immobilizing redox mediators directly onto the electrode surface to enhance electron transfer efficiency, potentially reducing the reliance on high-cost electrode materials like gold.

In summary, our study lays the groundwork for developing a bioelectrochemical cell for the anodic cultivation of *D. mccartyi* strain CBDB1 by identifying and characterizing cobalt chelates as putative anode mediators capable of shuttling electrons between RdhA of the OHR complex and an electrode. While cathodic reductive dehalogenation using cathode mediators has already been demonstrated in previous studies (Aulenta et al., 2007, 2010) offering significant potential for enhancing *in-situ* bioremediation of halogenated compounds, our approach is the first to show that electron flow in *D. mccartyi* cells can be coupled to an anode *via* redox mediators. The findings of this study provide a promising alternative to traditional cultivation methods for organohalide-respiring bacteria, eliminating the dependence on toxic organohalides for cultivation.

## Data Availability

The original contributions presented in the study are included in the article/Supplementary material, further inquiries can be directed to the corresponding author.
