# Supplementary material for: Reductive dehalogenase of Dehalococcoides mccartyi strain CBDB1 reduces cobalt- containing metal complexes enabling anodic respiration
Source: Front Microbiol. 2024 Oct 23;15:1457014. doi: 10.3389/fmicb.2024.1457014 (PMC11537884; doi:10.3389/fmicb.2024.1457014)
Supplement: Supplementary file 1 [file Data_Sheet_1.docx]

Supplementary material to the manuscript:

Reductive dehalogenase of Dehalococcoides mccartyi strain CBDB1 reduces cobalt-containing metal complexes enabling anodic respiration

# Supplementary data

## Supplementary Tables

**Supplementary TABLE** **1 | Turnover, described as the total number of electron acceptor molecules converted per second and cell (s^-1^ cell^-1^), of different substances used as putative anode mediators for the reductive dehalogenase (RdhA) in photometric methyl viologen-based *in-vitro* activity assays.** All compounds were used at a concentration of 300 µM. Activity assays were conducted in triplicates. The symbols + and – indicate whether a substance was reduced or not reduced by RdhA either abiotically (in the absence of *Dehalococcoides mccartyi* strain CBDB1 cells) or biotically. Turnover rates were calculated using the absorption changes at 630 nm, corrected for absorption changes observed in no-cell and no-substrate controls. n.d. – not determined.

| **ID** | **Electron acceptor** | **Reduction** | | **Turnover (s^‑1^ cell^‑1^)** |
| --- | --- | --- | --- | --- |
|  |  | **Abiotic** | **Biotic** |  |
| 1 | Methylene blue | – | – | n.d. |
| 2 | Neutral red | + | – | n.d. |
| 3 | Nile red | – | – | n.d. |
| 4 | Phenol red | – | + | 3.75 × 10^4^ |
| 5 | Indigo blue | – | + | 1.88 × 10^4^ |
| 6 | Selectofluor II | – | – | n.d. |
| 7 | 2-Chloro-1-methylpyridinium iodide | – | – | n.d. |
| 8 | 2,6-Dichloro-1-fluor-pyridinium-tetrafluoroborate | – | – | n.d. |
| 9 | *p*-Benzoquinone | – | – | n.d. |
| 10 | Tetramethyl-*p*-benzoquinone | – | – | n.d. |
| 11 | *p*-Chloranil | – | – | n.d. |
| 12 | Anthraquinone | – | – | n.d. |
| 13 | *o*-Naphthoquinone | – | – | n.d. |
| 14 | 2,5-Dibromo-6-isopropyl-3-methyl-*p*-benzoquinone | – | – | n.d. |
| 15 | Methyl-*p*-benzoquinone | – | – | n.d. |
| 16 | 2,3-Dimethoxy-5-methyl-*p*-benzoquinone | – | – | n.d. |
| 17 | 2,6-Dimethoxy-*p*-benzoquinone | – | – | n.d. |
| 18 | 2-Methyl-*p*-naphthoquinone | – | – | n.d. |
| 19 | 2,6-Bis(N-pyrazolyl)pyridine Ni(II) bromide | – | + | 2.10 × 10^4^ |
| 20 | Potassium oxalate Fe(III) chloride | – | + | 8.61 × 10^4^ |
| 21 | Cyanocob(III)alamin | – | + | 1.07 × 10^4^ |
| 22 | Methyl cob(III)alamine | – | + | 2.50 × 10^4^ |
| 23 | Methyl cobaloxime(III) | – | + | 1.90 × 10^4^ |

**Supplementary TABLE 2 | Peak potential, peak potential separation, peak current, and current peak ratios for methyl cob(III)alamin and methyl cobaloxime(III) were determined on different working electrode materials at various scan rates.** Data were calculated from cyclic voltammetry experiments using a platinum counter electrode, an Ag/AgCl reference electrode, and either gold or indium tin oxide as the working electrode. Abbreviations: *E*_pa_: oxidative/anodic peak potential, *E*_pc_: reductive/cathodic peak potential, Δ*E*_p_: peak potential separation, *i_pa_*: oxidative/anodic peak current, *i_pc_*: reductive/cathodic peak current. *i_pc_*/*i*_pa_: ratio of reductive to oxidative peak current.

| **Redox mediator** | **Scan rate (mV s^-1^)** | **Working electrode: gold** | | | | | | **Working electrode: indium tin oxide** | | | | | |
| --- | --- | --- | --- | --- | --- | --- | --- | --- | --- | --- | --- | --- | --- |
|  |  | ***E*_pa_** | ***E*_pc_** | **Δ*E*_p_** | ***i*_pa_** | ***i*_pc_** | ***i*_pc_/*i*_pa_** | ***E*_pa_** | ***E*_pc_** | **Δ*E*_p_** | ***i*_pa_** | ***i*_pc_** | ***i*_pc_/*i*_pa_** |
|  |  | **(mV)** | | | **(µA)** | |  | **(mV)** | | | **(µA)** | |  |
| **Methyl cob(III)alamin** | 25 | 284 | 192 | 92 | 0.29 | 0.26 | 0.90 | 253 | 156 | 97 | 0.05 | 0.04 | 0.80 |
|  | 50 | 284 | 202 | 82 | 0.23 | 0.20 | 0.87 | 258 | 126 | 132 | 0.05 | 0.03 | 0.60 |
|  | 100 | 284 | 202 | 82 | 0.52 | 0.41 | 0.79 | 263 | 161 | 102 | 0.09 | 0.08 | 0.89 |
|  | 250 | 294 | 202 | 92 | 0.86 | 0.66 | 0.77 | 263 | 146 | 117 | 0.12 | 0.09 | 0.75 |
|  | 500 | 304 | 202 | 102 | 1.21 | 1.01 | 0.83 | 274 | 156 | 118 | 0.17 | 0.17 | 1.00 |
|  | 750 | 304 | 202 | 102 | 1.57 | 1.43 | 0.91 | 263 | 151 | 112 | 0.22 | 0.18 | 0.82 |
|  | 1,000 | 304 | 202 | 102 | 1.99 | 1.68 | 0.84 | 263 | 161 | 102 | 0.16 | 0.14 | 0.88 |
|  | **Average** | **Δ*E*_p_** = 93 ± 8 mV ***i*_pc_/*i*_pa_** = 0.84 ± 0.05 | | | | | | **Δ*E*_p_** = 111 ± 11 mV ***i*_pc_/*i*_pa_** = 0.82 ± 0.12 | | | | | |
| **Methyl cobaloxime(III)** | 25 | 67 | -77 | 144 | 0.17 | 0.05 | 0.30 | -21 | -250 | 229 | 0.02 | 0.01 | 0.50 |
|  | 50 | 67 | -90 | 157 | 0.21 | 0.15 | 0.71 | -15 | -301 | 286 | 0.03 | 0.02 | 0.67 |
|  | 100 | 63 | -90 | 153 | 0.78 | 0.44 | 0.56 | 10 | -383 | 393 | 0.12 | 0.07 | 0.58 |
|  | 250 | 56 | -98 | 154 | 2.36 | 0.50 | 0.21 | -10 | -107 | 97 | 0.07 | 0.02 | 0.29 |
|  | 500 | 43 | -97 | 140 | 7.08 | 1.26 | 0.18 | n.d. | n.d. | n.d. | n.d. | n.d. | n.d. |
|  | 750 | 39 | -97 | 136 | 8.70 | 1.44 | 0.17 | n.d. | n.d. | n.d. | n.d. | n.d. | n.d. |
|  | 1,000 | 35 | -103 | 138 | 10.11 | 1.56 | 0.15 | n.d. | n.d. | n.d. | n.d. | n.d. | n.d. |
|  | **Average** | **Δ*E*_p_** = 146 ± 8 mV ***i*_pc_/*i*_pa_** = 0.33 ± 0.21 mV | | | | | | **Δ*E*_p_** = 251 ± 107 mV ***i*_pc_/*i*_pa_** = 0.51 ± 0.14 | | | | | |

**Supplementary TABLE** 3 **| Current densities of methyl cob(III)alamin and methyl cobaloxime(III) recorded on different working electrode materials and scan rates.** Cyclic voltammetry was performed using gold or platinum as the counter electrode and Ag/AgCl as reference electrode. Gold, with a surface area of 3.14 mm^2^, and indium tin oxide (ITO), with a surface area of 2.84 mm^2^, were used as working electrodes. Current densities (µA cm^-2^) were exemplary calculated at scan rates of 25 mV s^-1^ and 1,000 mV s^-1^ for the reduction and oxidation peaks.

| **Redox mediator** | **Scan rate (mV s^-1^)** | **Working electrode: gold Current density (µA cm^-2^)** | | **Working electrode: ITO Current density (µA cm^-2^)** | |
| --- | --- | --- | --- | --- | --- |
|  |  | **Reduction** | **Oxidation** | **Reduction** | **Oxidation** |
| Methyl cob(III)alamin | 25 | 6 | 7 | 1 | 2 |
|  | 1,000 | 54 | 63 | 5 | 6 |
| Methyl cobaloxime(III) | 25 | 10 | 19 | 1 | 1 |
|  | 1,000 | 104 | 324 | 1 | 3 |

**Supplementary TABLE 4| Calculation of the ellipsoid surface area in square Angstroms (in Å^2^) for various compounds, including halogenated compounds and anode mediators identified in this study, which can be reduced by the reductive dehalogenase from *Dehalococcoides mccartyi* strain CBDB1.** For each compound, the half-diameters (a × b × c) of the ellipsoid (in Å) were determined using ChimeraX. The surface area of the ellipsoids was calculated using the equation: $S\approx4\pi\left( \frac{a^{p}b^{p}+a^{p}c^{p}+b^{p}c^{P}}{3} \right)^{\frac{1}{p}}$ where *p* is an exponent typically chosen to be around 1.6075. Abbreviations used: MW – molecular weight, MeCoOx^III^ – methyl cobaloxime(III), MeCbl^III^ – methyl cob(III)alamin in the ‘base-on’ conformation, MeCbl^II^ – methyl cob(II)alamin in the ‘base-off’ conformation, BPB – Bromophenol blue, and 2,3,7,8-TCDD – 2,3,7,8-Tetrachlorodibenzo-*p*-dioxin.

| **Compound** | **Ellipsoid calculation** | **Sum formula** | **MW (g mol^-1^)** | **Ellipsoidal half-diameters (Å)** | **Surface area (Å²)** |
| --- | --- | --- | --- | --- | --- |
| MeCoOx^III^ | 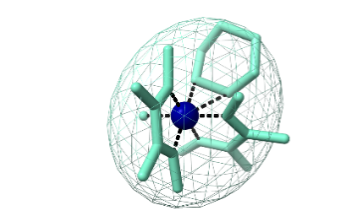 | C_14_H_22_CoN_5_O_4_ | 383.3 | 4.2 × 3.7 × 2.6 | 153.2 |
| MeCbl^III^ (base-on) | 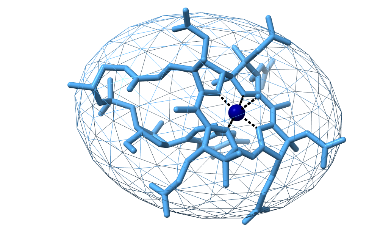 | C_63_H_91_CoN_13_O_14_P | 1344.4 | 9.2 × 7.1 × 5.0 | 626.4 |
| MeCbl^II^ (base-off) | 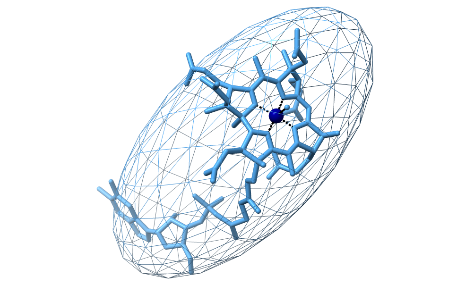 | C_63_H_91_CoN_13_O_14_P | 1344.4 | 14.0 × 6.8 × 5.0 | 869.8 |
| BPB | 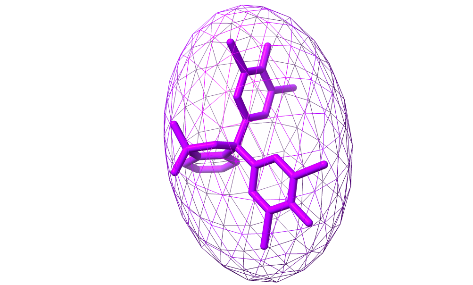 | C_19_H_10_Br_4_O_5_S | 670.0 | 7.4 × 5.1 × 3.8 | 364.5 |
| 2,3,7,8-TCDD | 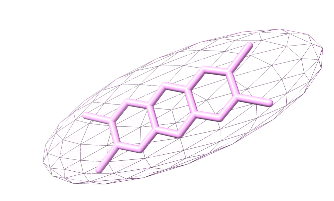 | C_12_H_4_Cl_4_O_2_ | 322.0 | 8.4 × 3.1 × 0.003 | 163.9 |

## Supplementary Figures


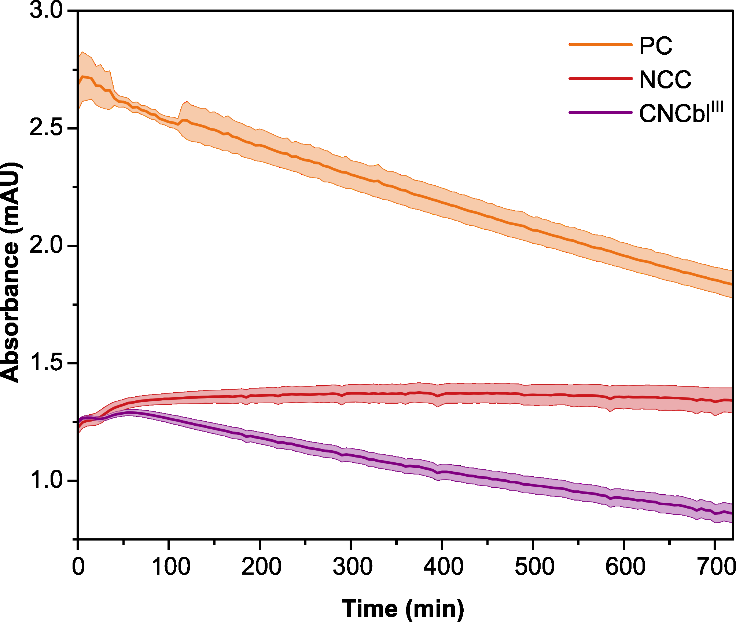


**Supplementary FIGURE** **1 |** **Absorbance changes at 630 nm over time in photometric methyl viologen-based *in-vitro* activity assays for the reductive dehalogenase (RdhA) from *Dehalococcoides mccartyi* strain CBDB1.** The activity assays were performed in five replicates, with the mean represented by a line and the standard deviation by the filled area under the line. The absorbance changes correlate with the re-oxidation and concentration of reduced methyl viologen in the presence of cyanocob(III)alamin (CNCbl^III^, purple line) as the electron acceptor. A positive control (PC, orange line) with 1,2,4,5-tetrachlorobenzene (TeCB), and a no-cell control (NCC, red line) containing CNCbl^III^ without CBDB1 cells were included.


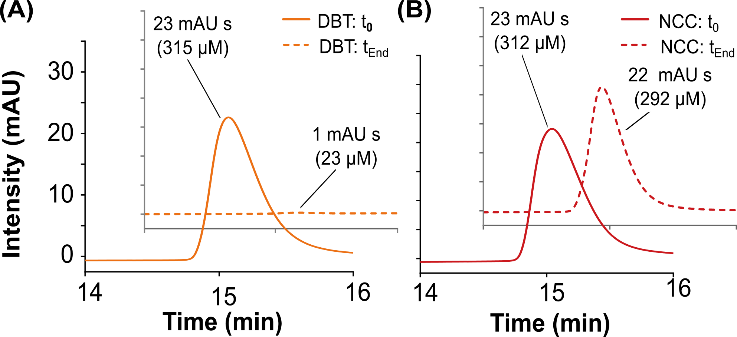


**Supplementary FIGURE** **2 | HPLC chromatograms of an activity assay with *Dehalococcoides mccartyi* strain CBDB1 cells using hydrogen as the electron donor and 3,5-dibromo-*L*-tyrosine (DBT) as the electron acceptor.** Chromatograms were recorded at the beginning of the reaction (t_0_) and after 24 hours (t_End_). **(A)** The elution peak of DBT, appearing at approximately 15 minutes, is shown at t_0_ (orange continuous line) and t_End_ (orange dashed line). **(B)** Chromatogram of the no-cell control (NCC) containing DBT at t_0_ (red continuos line) and t_End_ (red dashed line).


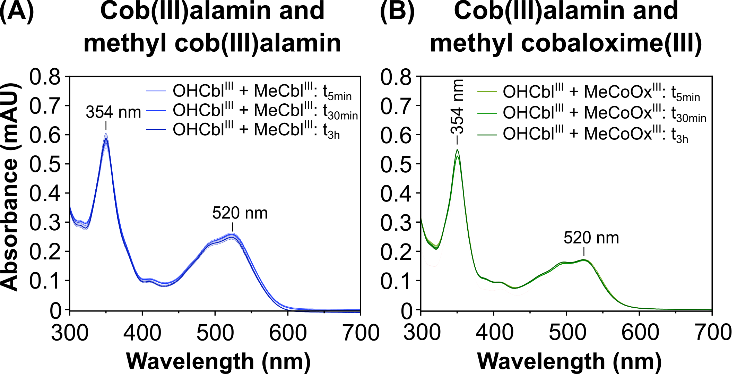


**Supplementary FIGURE** **3 | UV-Vis absorbance spectra of control assays containing hydroxocob(III)alamin (OHCob^III^) and the anode mediators methyl cob(III)alamin (MeCbl^III^) and methyl cobaloxime(III) (MeCoOx^III^).** Spectra were recorded after 5 minutes (t_5min_), after 30 minutes (t_30min_) and after 3 hours (t_3h_). Samples containing anode mediators were set up in five replicates, controls without mediators in triplicates. **(A)** Absorbance spectra of assays containing OHCob^III^ and the mediator MeCbl^III^ (blue lines) at t_5min_, at t_30min_, and t_3h_. **(B)** Absorbance spectra of assays with OHCob^III^ and the mediator MeCoOx^III^ (green lines) at t_5min_, t_30min_, and t_3h_.


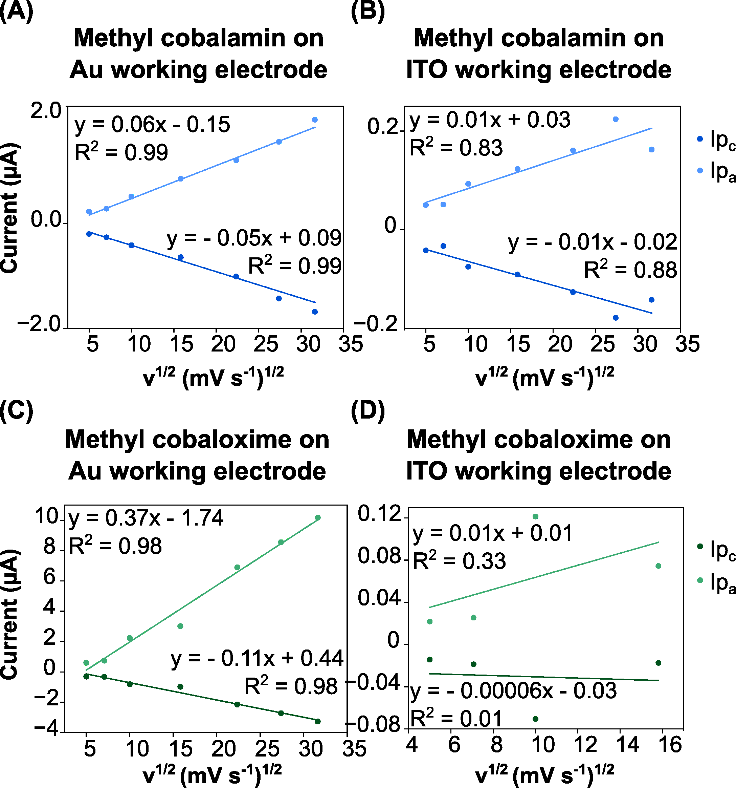


**Supplementary FIGURE 4 | Correlation of the cathodic (*i*_pc_) and anodic (*i*_pa_) peak currents with the square root of the scan rate for methyl cob(III)alamin (A, B) and methyl cobaloxime(III) (C,D).** Cathodic and anodic peak currents were determined *via* cyclic voltammetry using gold or platinum as the counter electrode, Ag/AgCl as the reference electrode, and either gold (A, C) or indium tin oxide (ITO) (B, D) as the working electrode. Linear regression analysis was performed using OriginLab.


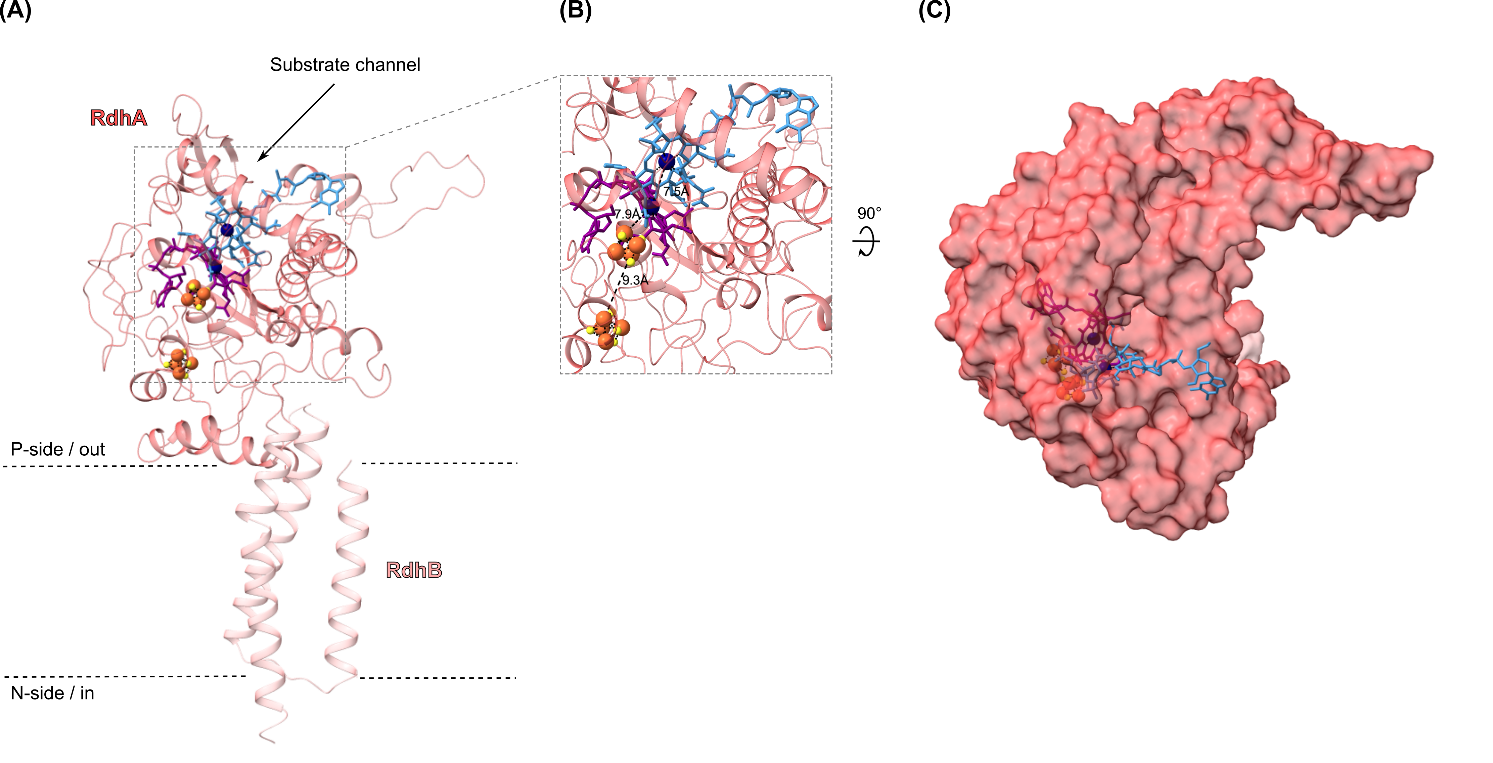


**Supplementary FIGURE** **5 | *In-silico* structure of the reductive dehalogenase RdhA (locus cbdbA84) and its anchoring protein RdhB (cbdbA85) from *Dehalococcoides mccartyi* strain CBDB1, predicted with AlphaFold2, including docking of the putative anode mediator methyl cob(III)alamin at the enzyme’s active center. (A)** Cartoon representation of RdhA (dark pink) and RdhB (light pink), computed with the AlphaFold2 ColabFold platform, embedded into the cytosolic membrane. The binding of the [4Fe-4S] clusters (yellow-orange spheres) and the corrinoid cofactor (purple stick conformation) was defined based on the active center of the *Dh*PceA subunit from *Desulfitobacterium hafniense* strain TCE1. Docking of methyl cob(III)alamin (blue stick conformation) was performed using AutoDock Vina. Cobalt ions of the corrinoid cofactor and methyl cob(III)alamin are shown in dark blue spheres. **(B)** Zoom-in of the active center of RdhA with methyl cob(III)alamin docked to the corrinoid cofactor. The distances between the metallocofactors and the putative anode mediator are shown in Angstroms (Å). **(C)** Surface structure of the RdhAB module of strain CBDB1, rotated 90° to the front around the x-axis to show the substrate channel in RdhA from the top perspective.
